# An improved method for whole-mount *in situ* hybridization in regenerating tails of *Xenopus laevis* tadpoles

**DOI:** 10.3389/fcell.2024.1487644

**Published:** 2024-12-09

**Authors:** A. D. Shitikov, E. A. Parshina, A. G. Zaraisky, M. B. Tereshina

**Affiliations:** ^1^ Shemyakin-Ovchinnikov Institute of Bioorganic Chemistry, Russian Academy of Sciences, Moscow, Russia; ^2^ Department of Regenerative Medicine, Pirogov Russian National Research Medical University, Moscow, Russia

**Keywords:** whole-mount *in situ* hybridization, photobleaching, tail regeneration, refractory period, *Xenopus laevis*, myeloid cells, MMP9

## Abstract

Whole-mount *in situ* hybridization (WISH) is a widely used method that supports the concept of “seeing is believing” by enabling the visualization of gene expression patterns in whole-mount multicellular samples or sections. This technique is essential in the study of epimorphic regeneration in cold-blooded vertebrates, where complex three-dimensional organs such as tails, limbs, and eyes are completely restored after loss. The tadpoles of the frog *X. laevis* serve as a convenient model for studying regeneration, as they can regenerate their tails within a week after amputation. Modern high-throughput sequencing methods have identified various cell populations involved in the regeneration process and determined the repertoire of genes activated during this time. Specifically, a population of reparative myeloid cells expressing *mmp9* as a marker gene has been shown to be crucial for the initial stages of tail regeneration in *X. laevis* tadpoles. The validation of these data and further examination using WISH offers the advantage of providing detailed information on the spatial and temporal dynamics of target gene expression levels. However, detecting mRNA by WISH can be challenging when mRNA levels are very low, transcripts are localized in hard-to-access areas, or tissue samples are prone to background staining, as is the case with *X. laevis* regenerating tail samples. Here, we describe additional treatments for regenerating tail samples that minimize background staining and enhance the visualization of cells containing target RNA through *in situ* hybridization. Using an optimized WISH protocol on *X. laevis* tadpole tail regenerates, we obtained novel data on the *mmp9* expression pattern during the first day post-amputation at the regeneration-competent stage 40 and the regeneration-incompetent stage 47 (refractory period). The significant differences in the expression patterns indicate that *mmp9* activity is positively correlated with regeneration competence.

## 1 Introduction

One of the most frequently used methods in the concept of “seeing is believing” is *in situ* hybridization, which allows the visualization of the spatio-temporal expression pattern of a gene of interest in the cells of whole organisms or tissues (Herrmann and Neidhardt, 2006). This method involves the hybridization of a labeled antisense RNA probe with the corresponding endogenous mRNA in the sample, followed by a label-determined staining step. The importance of this method among developmental molecular biologists cannot be overstated.

Despite the emergence of high-throughput methods such as single-cell or bulk RNA sequencing, spatial transcriptomics, and proteomics, the results obtained by *in situ* hybridization remain a staple in many publications, providing crucial validating data. However, detecting mRNA by WISH can be challenging when mRNA expression levels are very low, the transcripts are localized in hard-to-reach or hard-to-view areas, or tissue samples are prone to background staining, which decreases the signal-to-noise ratio.

One of such case is WISH in regenerating tail of *Xenopus* frog tadpoles, which can regenerate an amputated tail in a week and a hind limb in a month (Beck C.W. 2012). This model along with other models of fins, tails and limbs regeneration in fishes and amphibians is widely used to study mechanisms of regeneration in amniotic species, which are distinguishing by their amazing ability to restore these body appendages (Murawala et al., 2012). The study of genes associated with regeneration in model anamniotic species is of great interest not only for basic science, but also for the development of approaches that could enhance regeneration in amniotes, especially in human. The point is that amniotes (reptiles, birds and mammals) cannot regenerate body appendages because such important stages of regeneration as the formation of the regenerative epithelia and blastema cannot be realized in them. In particular, this is attributed by the destruction of gene network and loss of many genes responsible for these processes occurred during amniotes evolution (Zaraisky et al., 2024).

Here we describe additional treatments for *Xenopus laevis* tadpoles' regenerating tail samples, which allow us to obtain clear, high-contrast images of target RNA-containing cells by WISH. The most challenging aspect of this method is minimizing background staining in the sample to achieve high-sensitivity detection of cells expressing the target gene. For wild-type *X. laevis* tadpole tails at different stages of regeneration, this presented a double challenge.

Firstly, melanosomes (pigment granules) actively migrate with cells to the amputation site and can therefore interfere with the BM Purple stain signal. Additionally, due to the numerous melanophores, visualization and photodetection of the staining signal are very difficult (El Mir et al., 2024). If melanophores are not the focus of the investigation, we suggest adding a bleaching step for the tadpoles, which has proven effective for decoloring both melanosomes and melanophores after fixation and before the pre-hybridization stages.

Secondly, tail fins are very loose tissues, and the main problem during *in situ* hybridization is strong background staining, especially when the target RNA is not highly expressed and requires long staining incubation. We have observed that tadpole samples fixed immediately after amputation (0 hpa, hours post amputation) exhibited the lowest background staining of fins. To address the background problem, we recommend making fin incisions in a fringe-like pattern at some distance from the area of interest in the regenerating tail. This tail fin notching procedure improved the washing out of all solutions, preventing BM Purple from getting trapped in the loose fin tissues and causing non-specific autocromogenic reactions. Even after 3–4 days of staining, no background staining was detected. The combination of these procedures before hybridizing the regenerating tail samples allowed us to obtain high-contrast images of gene expression patterns without background interference.

To test various WISH protocols, we chose the gene encoding Zn2+-dependent extracellular matrix metalloproteinase 9 (*mmp9*), whose expression pattern was firstly detected in *X. laevis* albino embryos and tadpoles during normal development and hindlimb bud and lens regeneration (Carinato et al., 2000). Recently *mmp9* was identified as one of the genes specific to the population of reparative myeloid cells that plays a key role in tail regeneration in *X. laevis* tadpoles (Aztekin et al., 2019; Aztekin et al., 2020). This lineage of cells is essential for the early steps of regeneration, as it quickly replaces the inflammatory myeloid lineage and induces subsequent processes necessary for regeneration progression: apoptosis and tissue remodeling, which ultimately lead to the relocalization of regeneration-organizing cells responsible for progenitor proliferation (Aztekin et al., 2019; Aztekin et al., 2020). According to yet unpublished data, *mmp9*, along with *mpepa1*, *junb*, *mmp1* and *mmp8*, are unique markers of regeneration-inducing cells (RICs), which are formed transiently from basal epidermal cells. These markers are critical for modifying the surrounding extracellular matrix to facilitate the migration of other cell types, such as regeneration-organizing cells that further promote regeneration. These markers were identified through bulk, single-cell, and spatial RNA-seq (Radek et al., 2023).

As a result, we demonstrate, for the first time to our knowledge, the detailed expression pattern of *mmp9* during the early stages (0, 3, 6, and 24 h post-amputation) of tail regeneration in tadpoles at stage 40. Additionally, we show that the activity of *mmp9* is closely linked to regeneration competency. Its expression pattern significantly differs in tails amputated during the refractory period (stages 45–47), when regeneration is temporarily blocked as part of the normal developmental program (Beck et al., 2003). The high-quality images of regenerating tails, stained for *mmp9*-expressing cells using our optimized WISH protocol, allowed us to observe the behavior of these cells during the early stages of regeneration and to clarify and supplement data obtained by high-throughput methods, such as bulk- and sc-RNAseq.

## 2 Design of experiments

**FIGURE 1:**
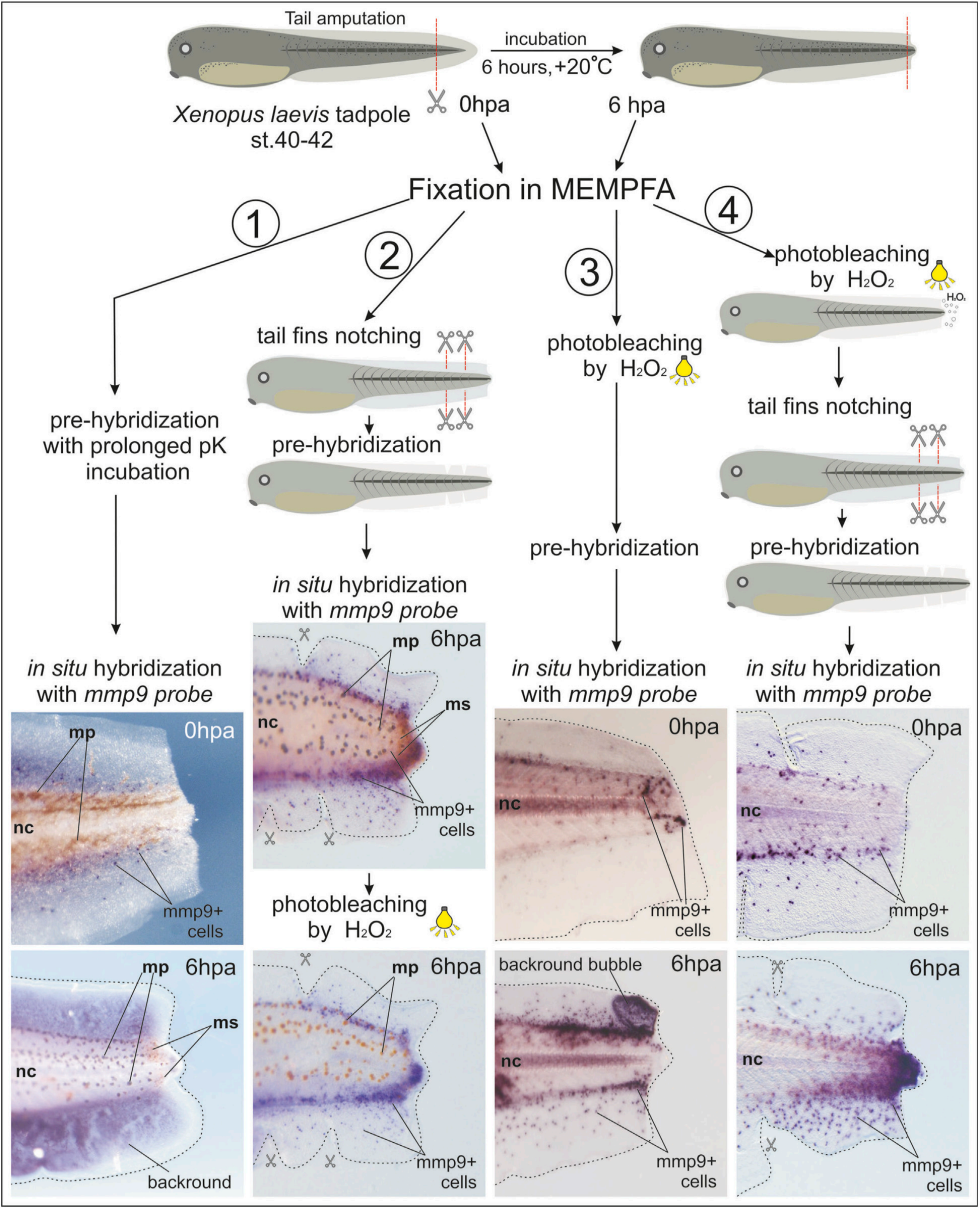
Comparison of different treatments for whole-mount samples of regenerating tails of *Xenopus laevis* tadpoles to obtain a clearer *in situ* hybridization signal from *mmp9*+ cells. 1st variant: Prolonged proteinase K (pK) incubation during the pre-hybridization step, which did not improve staining signal clarity or reduce background staining. 2nd variant: Combination of tail fin notching before WISH and photo-bleaching after BM Purple staining. 3rd variant: Addition of a photo-bleaching step before WISH. 4th variant: Early photo-bleaching after fixation in MEMPFA and rehydration, followed by tail fin notching. This variant provided the clearest image results. Mp–melanophores, ms–melanosomes, nc–notochord.

To optimize the *in situ* hybridization staining in the regenerating tails of *X. laevis* tadpoles, we tested several additional treatments, as shown in Figure 1. We used tadpoles at stage 40 with tails regenerating for 0 or 6 hpa to evaluate different protocol variants. Samples at 0 and 6 hpa (12–15 tadpoles) for each protocol variant were collected in at least three independent experiments.

Treating samples with proteinase K helps to remove nucleases and makes the tissue more permeable to reagents. Lengthening the incubation time with proteinase K solution for samples at later developmental stages can increase the sensitivity of *in situ* hybridization and reduce non-specific staining.

In the first variant, we extended the proteinase K incubation time for tadpole tail samples to 30 min, but the staining results were unimpressive, with *mmp9*+ cells overlapping with strong background staining.

In the second variant, we partially notched the edges of the fin to help wash out reagents causing background staining from the loose tissues. This approach allowed us to observe many more *mmp9*+ cells. To reduce overlapping of the BM Purple stain with melanosomes and melanophores, we added a post-staining photo-bleaching step, as recommended by Harland (1991). The result was improved imaging, but melanophores only faded to brown.

In the third variant, we moved the photo-bleaching step to the very beginning of the protocol, immediately after fixation in MEMPFA and the dehydration step. This resulted in perfectly albino tails, but some samples developed large bubbles in the tail fin area filled with non-specific BM Purple staining after *in situ* hybridization.

Finally, in the fourth variant, we combined the early photo-bleaching step with caudal fin cutting before sample hybridization, resulting in very clear images of the specific staining of *mmp9*+ cells. The step-by-step procedures for this optimized protocol are described below.

### 2.1 Materials

1. MEMPFA solution for samples fixation.

**Table:** 

MEMPFA		
Reagent	Final concentration	Amount
Paraformaldehyde (PFA)	4%	4 g
EGTA (0.5 M)	2 mM	200mkl
MgSO₄ (1 M)	1 mM	100mkl
MOPS (1 M)	100 mM	10 mL
ddH₂O	N/A	89.7 mL
**Total**	N/A	**100 mL**
Adjust pH to 7.4		

Procedure for preparing MEMPFA:

First add PFA powder to half the total volume of water, add NaOH (approximately 100 µL per 100 mL) and heat to 60°C for better dissolution. Once the PFA is completely dissolved, cool the solution to room temperature, add the remaining reagents and adjust the pH to 7.4.

**Note**: MEMPFA solution stored at +4°C can be used to fix samples for 2 weeks. It can subsequently be used for postfixation and storage of samples after *in situ* hybridization.

2. The *Xenopus* embryos and tadpoles are incubated in MMR 0,1x with Hepes 1x.

**Table:** 

MMR 0,1x solution for *Xenopus* embryos/tadpoles incubation		
Reagent	Final concentration	Amount
MMR 20x	0,1x	5 mL
HEPES 200x	1x	5 mL
ddH₂O	N/A	990 mL
**Total**	N/A	**1,000 mL**
MMR 20x		
NaCl	2 M	58.45 g
KCl	38 mM	1.43 g
CaCl₂*2H₂O	40 mM	2.94 g
MgCl₂*6H₂O	42 mM	2.03 g
ddH₂O	N/A	500 mL
**Total**	N/A	**500 mL**
Hepes 200x		
**Hepes**	1 M	23.83 g
**ddH₂O**	N/A	100 mL
**adjust pH to 7.4 with NaOH**		

3. PBS 1x solution for washes was prepared from PBS 20x stock. PTW solution was prepared from PBS 1x by addition of Tween20 detergent till 0.1% final concentration.

**Table:** 

PBS 20x		
Reagent	Final concentration	Amount
**NaCl**	120 mM	70.13 g
Na₂HPO₄*7H₂O	7 mM	18.8 g
NaH₂PO₄*2H₂O	3 mM	4.7 g
**KCl**	2.7 mM	2 g
**ddH₂O**	N/A	450 mL
**Total**	N/A	**500 mL**
PTW		
PBS 20x	1x	25 mL
Tween-20 (100%)	0.1%	0.5 mL
**Total**	N/A	**500 mL**

4. Pre-Hybridization Buffer and its ingridients.

**Table:** 

PH-buffer		
Reagent	Final concentration	Amount
Formamide (100%)	50%	10 mL
SSC solution (20x)	5x	5 mL
Tortula RNA (50 mg/g)	1 mg/mL	400 mkl
Denhardt’s solution (50x)	0.02%	400 mkl
Tween-20 (100%)	0.1%	20 mkl
Chaps (10%)	0.1%	200 mkl
EDTA (0.5 M)	10 mM	400mkl
ddH₂O	N/A	3.58 mL
**Total**	N/A	**20 mL**
Adjust pH to 7.4		

**Note:** When preparing the PH buffer use RNase-free tubes, pippets, tips and wear gloves. RNase contamination can lead to dig-RNA degradation in the hybridization probe and *in situ* hybridization failure. The PH-buffer can be stored at −20^o^C in tube with parafilme-wrapped lid.

**Table:** 

Denhardt’s solution 50x		
Reagent	Final concentration	Amount
Ficoll	1%	0.5 g
Polyvinylpyrrolidone	1%	0.5 g
BSA	1%	0.5 g
ddH₂O	N/A	50 mL
**Total**	N/A	**50 mL**

Stock Denhardt’s solution (50x) can be stored at −20^o^C.

**Table:** 

SSC solution 20x		
Reagent	Final concentration	Amount
NaCl	3 M	8.75 g
C₆H₅NaO₇*2H₂O	0.34 M	5 g
ddH₂O	N/A	50 mL
**Total**	N/A	**50 mL**
adjust pH to 7.4 with HCl		

Stock solution can be stored at −20^o^C. SSC 2x and 0,2x solutions for washing off dig-probe are prepared from stock solution by dilution 10 or 100 times with double distilled water.

5. MAB solution for incubation with anti-bodies and further washing off.

**Table:** 

MAB		
Reagent	Final concentration	Amount
Maleic acid	100 mM	5.9 g
NaCl	150 mM	4.35 g
ddH₂O	N/A	500 mL
**Total**	N/A	**500 mL**
adjust pH to 7.4 with NaOH		

Prepare a fresh MAB solution and use it for one WISH cycle, storing at +4C between incubation and antibody washout.

6. Alkaline phosphatase buffer is prepared fresh with an endogenous AP inhibitor (levamisole) for preparation for staining and without it for washing.

**Table:** 

Alkaline phosphotase buffer (AP-Buffer)		
Reagent	Final concentration	Amount
Tris-HCl, pH = 9.5	100 mM	10 mL
MgCl₂ (2 M)	50 mM	2.5 mL
NaCl (5 M)	100 mM	2 mL
Tween-20 (100%)	0.1%	500 mkl
Levamisole (500 mM)	2 mM	400 mkl
ddH₂O	N/A	84,6 mL
**Total**	N/A	**100 mL**

7. The BM Purple substrate (Roche, MERCK) is used with addition of levamisole till 1 mM final concentration.

### 2.2 Equipment

The following equipment was used in the current work:

Applied biosystems ProFlex PCR system (Thermo Fisher Scientific), thermostate “GNOM” (DNA-technologies), Eppendorf MiniSpin centrifuge, Leica KL300 LED binocular, thermostate incubator (Thermo Fisher Scientific), Vannas scissors, orbital shaker OS-20 (Biosan), hot shaker (Bellco Biotechnology), Leica binocular M205 with camera Leica DC 400F, fluorescent lamp light (OSRAM L 18W/77), cryotome MICROM HM 525 (Thermo Scientific).

## 3 Step-by-step procedures

Our laboratory has historically used the *in situ* hybridization protocol described by Richard Harland (Harland et al., 2004) as it is optimized for *X. laevis* embryos. However, in regenerating tail samples, this protocol resulted in strong background staining of the tail fins. Below, we describe our additional sample treatments and modifications to the *in situ* protocol for regenerating tadpole tail samples that significantly improved the signal-to-noise ratio.

The first step in preparing for an *in situ* hybridization experiment is to clone the gene of interest and synthesize the corresponding antisense RNA labeled with dig-/fluorescein-/dnp-uridine residues and prepare a hybridization probe (Long and Rebagliati, 2002). Hybridization with labeled sense RNA, which does not bind to the RNA of interest and allows detection of the presence of basic background staining, was used as a control.

### 3.1 *Mmp9* cloning, dig-RNA synthesis and dig-probe preparation

1) The fragment of *mmp9. S* cDNA (GenBank accession number NM_001086503) was amplified using PCR method. The DNA matrix was presented by the cDNA reversely transcribed by MMLV-RT (Evrogen) from mRNA of 1dpa (days post amputation) regenerating tails of *X. laevis* tadpoles extracted by RNAextract reagent (Evrogen). The following primers were used: *mmp9. S* F 5’ – TTC​ACT​CGT​ATA​TAC​AGC; *mmp9. S* FTest 5’ - TAT​CTT​CGA​CGG​AGT​GTC​AT and *mmp9. S* R 5’ – TGC​ACA​TCA​CTG​TGA​TCC​A. After purificatioin the PCR-fragment was cloned into pAL2-T vector (Evrogen) and then several clones were sequenced. On the base of correct clone the PCR-fragment of *mmp9* with SP6 promoter in the reverse orientation was obtained and purified with elution in RNAse-free water (Encyclo plus PCR kit, CleanUp Standard, Evrogen).
2) The Dig-labeled RNA antisense probe for the whole-mount *in situ* hybridization was synthesized by SP6 polymerase from SP6 transcription kit (mMessage mMachine, Thermo Fisher Scientific) with DIG RNA Labeling Mix (Roche) and purified *mmp9* PCR-product obtained with *mmp9. S* F and M13 reverse primers as a matrix. The dig-labeled sense-RNA for *mmp9* was synthesized by T7 polymerase from T7 transcription kit (mMessage mMachine, Thermo Fisher Scientific) with DIG RNA Labeling Mix (Roche) and purified *mmp9* PCR-product obtained with M13 forward and *mmp9. S* R primers as a matrix. The obtained dig-RNAs were purified by CleanRNA Standard kit (Evrogen) and stored at −20^o^C.
3) To prepare *mmp9* dig-RNA probe 1000 ng of antisense or sense dig-RNA was diluted in 1 mL of pre-hybridization buffer (PHB). The probe can be stored at −20^o^C.

**Note:** The probe can be re-used 2–3 times, on the 4th time we usually add 500 ng of dig-RNA to 1 mL of old probe and use it 2-3 more times.

### 3.2 Tadpoles tail amputation, fixation and dehydration

4) Tadpoles were incubated in 0,1xMMR till the 40–41 stage or 45–46 according to normal tables of *Xenopus* development (Zahn et al., 2022). Before amputation tadpoles were placed into Petri dish covered with 2% agarose and filled with anesthetic solution. The quarter of tail was amputated by Vannas scissors and 0 hpa tadpoles were replaced into fresh 0,1MMR solution and have been incubated for 6 h at room temperature.
5) The anesthetized regenerating tadpoles were placed in 11 mL-glass vials containing 5–7 mL of cold, freshly prepared MEMPFA solution and incubated overnight at 4^o^C. All incubations are hold on the slightly rotating (<60 rpm) platform of orbital shaker at room temperature or in incubated orbital shaker.
6) Afterwards the MEMPFA solution was replaced by 1xPBS (5–7 mL) and washed three times for 5–10 min at RT (room temperature).
7) The graded dehydratation of samples was made by washing with 5–7 mL of the following solutions at RT on the rotating platform:
a) 25% EtOH/75% PBS – 5–10 min
b) 50% EtOH/50% PBS - 5–10 min
c) 75% EtOH/25% PBS -5–10 min
d) 96% EtOH - 5–10 min
e) 96% EtOH -storage

**II Pause point**. Dehydrated samples can be stored at −20°C for several months until the inspiration arises to perform *in situ* hybridization.

### 3.3 Tadpoles photobleaching and post-fixation

In the case of albino tadpoles, the bleaching step can be skipped. However, if only wild-type tadpoles are available, the problem of accumulating near the amputation line melanosomes (granules of embryonic pigment) and melanophores (black pigment cells) complicating the visualization of *in situ* hybridization staining arises. Here we describe the optimized bleaching protocol for regenerating tails samples.

**Note:** Wear gloves and lab coat.

8) Replace 96% EtOH with bleaching solution – 3%H₂O₂ in 96%EtOH.

**Note**: the bleaching solution must be freshly prepared.

9) Lie down glass vials with samples in bleaching solution on a tray lined with foil. Place the tray on the orbital shaker under the fluorescent lamp light (OSRAM L 18W/77) and bleach for 12–24 h.
10) Wash samples twice by 5 mL of 96% EtOH.
11) Rehydrate samples by sequential washes
a) 5 mL 75% EtOH/25% PBS 5–10 min
b) 5 mL 50% EtOH/50% PBS 5–10 min
c) 5 mL 25% EtOH/75% PBS 5–10 min
d) 5 mL 1xPBS 5–10 min
e) 5 mL 1xPBS 5–10 min
12) Post-fixate samples in 5 mL MEMPFA for 1 h at room temperature.

**Note**: We found that melanophores are discolored best of all if bleached before the WISH pre-hybridization, but the melanosomes can be bleached before as well as after WISH procedures (see images on Figure 1).

### 3.4 Tail fin notching

The background staining of tail fins makes it difficult to distinguish weak target staining. We hypothesized that the background staining in long-lasting colorings is due to the loose structure of fin tissues, which obstructs effective washing out of non-specific hybrids, unbound probes, and staining solutions, leading to non-specific BM Purple staining. To minimize this issue, we attempted to increase the time of proteinase K treatment, but this had no effect. Previous results have shown that *mmp9* is expressed primarily in the peri-trunk area at stage 40 (Carinato et al., 2000). Therefore, we made 3-4 cuts on the upper and 2-3 cuts on the lower tail fins using Vannas scissors or an ophthalmic scalpel. The distance between cuts was 600–800 μm, and the depth of the cut corresponded to the width of the fin from the outer edge to the trunk muscles (in the range of 200–300 μm for the upper and 400–600 μm for the lower fin). We do not recommend cutting the muscle tissue, as it leads to a stronger deformation of the sample after hybridization procedures and sometimes causes background staining of the edges of the cut muscle tissue, which makes it difficult to visualize the true-positive signal.

13) Wash samples with 5 mL of PBS three times for 5–7 min each. Place tadpoles in an agarose-coated Petri dish with PBS. Make several cuts in the tail fins under a binocular microscope. Return the tadpoles to vials and process according to the WISH protocol optimized in our laboratory for *X. laevis.*

### 3.5 Whole-mount *in situ* hybridization

#### 3.5.1 Pre-hybridization

All washes/incubations are hold on the slightly rotating (<60 rpm) platform of orbital shaker by default at room temperature unless otherwise specified.

14) Remove PBS and add 5 mL of pK-solution (recommended working concentration of Proteinase K is 1 mkg/mL), incubate 10–15 min
15) Change solution on Glycin solution (working concentration is 2 mg/mL) incubate for 10 min
16) Wash twice with 5 mL PTW for 5–7 min
17) Remove PTW and add 3 mL of MEMPFA for post-fixation during 20 min
18) Do two washes by 5 mL PTW for 5–7 min each
19) Remove PTW and add 4 mL of PTW and 1 mL of Pre-hybridization buffer (PHB), incubate for 15 min
20) Set change solution on 1 mL of PHB and incubate at +60^o^C.

**Note**: *X. laevis* embryos at early developmental stages (till st.25) can be moved to +60°C after room temperature without any harm, but the tadpoles may curl up, making imaging awkward. To minimize distortion, we gradually increase the temperature in the incubated orbital shaker, starting from +35°C to +60°C with two 10°C intervals and one 5°C interval. We maintain incubation at each temperature level for 20–30 min. Finally, the samples are incubated in PHB at +60°C for at least 30 min.

21) Change PHB on fresh pre-heated at +60^o^C 0.5 mL PHB and incubate vials at +60^o^C for 4–16 h.

#### 3.5.2 Dig-RNA-hybridization

22) Replace the PHB with a 0.5 mL dig-RNA probe preheated at +60°C and incubate for 12–15 h in an orbital shaker at +60°C.

#### 3.5.3 Dig-probe washing out and incubation with antibodies

23) Carefully remove dig-probe with a pipette into eppendorf, wrap in parafilm and store at −20 for re-use. Add pre-heated at +60^o^C 0.5 mL PHB. Incubate 30 min at +60^o^C.
24) Remove PHB and add pre-heated at +60^o^C 0.5 mL PHB, incubate 30 min at +60^o^C.
25) Wash twice with 5 mL of pre-heated at +60^o^C 2x SSC, incubate 1 h at +60°C.
26) Remove 2xSSC, add 5 mL of pre-heated at +60^o^C 0,2xSSC and transfer vials on the orbital shaker at room temperature, incubate 30 min. All further incubations are hold at room temperature.
27) Wash with 5 mL 0,2xSSC for 30 min.
28) Change 0,2xSSC on 5 mL MAB and incubate 10 min.
29) Wash with 5 mL MAB for 10 min.
30) Remove MAB, add 1 mL of pre-Blocking Solution (without calf serum) (final concentration of blocking reagent is 2%) and incubate 20 min.
31) Remove pre-Blocking solution, add 1 mL Blocking solution (+20% Heat inactivated calf serum) and incubate 1–2 h.
32) Change solution on 0.5 mL Blocking solution +0.1% anti-dig-AP antibodies and incubate at +4°C overnight on the orbital shaker.

#### 3.5.4 Anti-bodies wash, chromogenic staining and post-fixation

33) Remove solution with antibodies, add 5 mL MAB and incubate 1 h. Make in sum 5 washes with MAB.

**II Pause point:** The final rinse can be left in the refrigerator overnight.

34) Prepair fresh AP-Buffer with levamisole, inhibitor of endogenous alkaline phosphatase. Change MAB to 3 mL of AP Buffer (lev+), incubate 15–20 min.
35) Repeat AP buffer (lev+) rinse for 15–20 min.
36) Add 1mkl levamisole into 0.5 mL of BM Purple, change AP buffer to BM Purple (lev+) and place the vials into dark room/box/wrap into foil. Incubate at RT till appropriate staining. If necessary the vial(s) may be placed into refrigerator and left overnight, the rate of staining at +4^o^C is very low. It is better to change the BM Purple (lev+) if it starts to turn blue.

**Note**: BM Purple is sensitive to the light and must be kept in darkness, otherwise it starts autochromogenic reaction with multiple colored sediments, sticking on the samples.

37) Upon reaching the required color intensity, replace BM Purple with AP buffer (lev-), rinse twice with 2–3 mL for 15–20 min.
38) Change AP buffer to MEMPFA for staining fixation.

**Note**: Of course, like all methods, this one has its own sensitivity limits. According to our experience, to detect genes that are revealed in transcriptome sequencing (https://www.ncbi.nlm.nih.gov/geo/query/acc.cgi?acc=GSE88975 and our unpublished data, platform Illumina 2000) in the amount of 70–170 normalized by DESeq counts, staining step should be continued for 3–4 days, putting the samples in the refrigerator overnight and changing the BM Purple solution to a fresh one as it turns blue. The background staining can grow in the notochord, but at the same time the pattern of the gene of interest gradually appears. If it is not diffuse, but concentrated in certain cells or their clusters, then the pattern will be clearly visible. Staining of genes with over 500 counts requires 1–2 days of development (for *mmp9* at 24hpa 890, our unpublished data), over 3,000–4–5 h.

#### 3.5.5 Samples imaging and storage

39) To photograph the samples wash them with PBS three times and make images on agarose-coated Petri dish with PBS under the stereomicroscope (in our case it was Leica M205) with camera (Leica DC 400F).
40) For storage change PBS to MEMPFA and store at +4^o^C or perform graded dehydration as in step 4) and store in EtOH 96% at −20^o^C.
41) For more detailed pattern describing the samples after *in situ* hybridization can be cryosectioned, treated with dapi and embedded into mowiol for imaging.

## 4 Results and discussion

Although the expression activity of *mmp9* during early *Xenopus* development, wounding, and hindlimb regeneration was demonstrated as early as 2000 (Carinato et al., 2000), the first data on its spatiotemporal expression pattern in the regenerating tadpole tail tip appeared relatively recently. However, these data are quite contradictory and not complete. For the first time, *mmp9* expression during *Xenopus* tadpole tail regeneration was reported at 1, 2, and 3 dpa in reparative myeloid cells using scRNA-seq (Aztekin et al., 2019). As the authors of this study showed, after amputation at stage 40 (0 dpa), the number of *mmp9* transcript counts decreased up to 1 dpa with a subsequent increase to 3 dpa. However, in the work of other authors, an increase in *mmp9* expression over background level was detected using bulk HiSeq already at 0.5-1 hpa, followed by further rise in expression up to 6 hpa and a subsequent drop down to 3 dpa (Radek et al., 2023). These authors also validated *mmp9* expression by *in situ* hybridization and spatial transcriptomics only at 1 dpa, and both methods showed rather blurred images of expression regions along the trunk and on the amputated tail tip.

**FIGURE 2:**
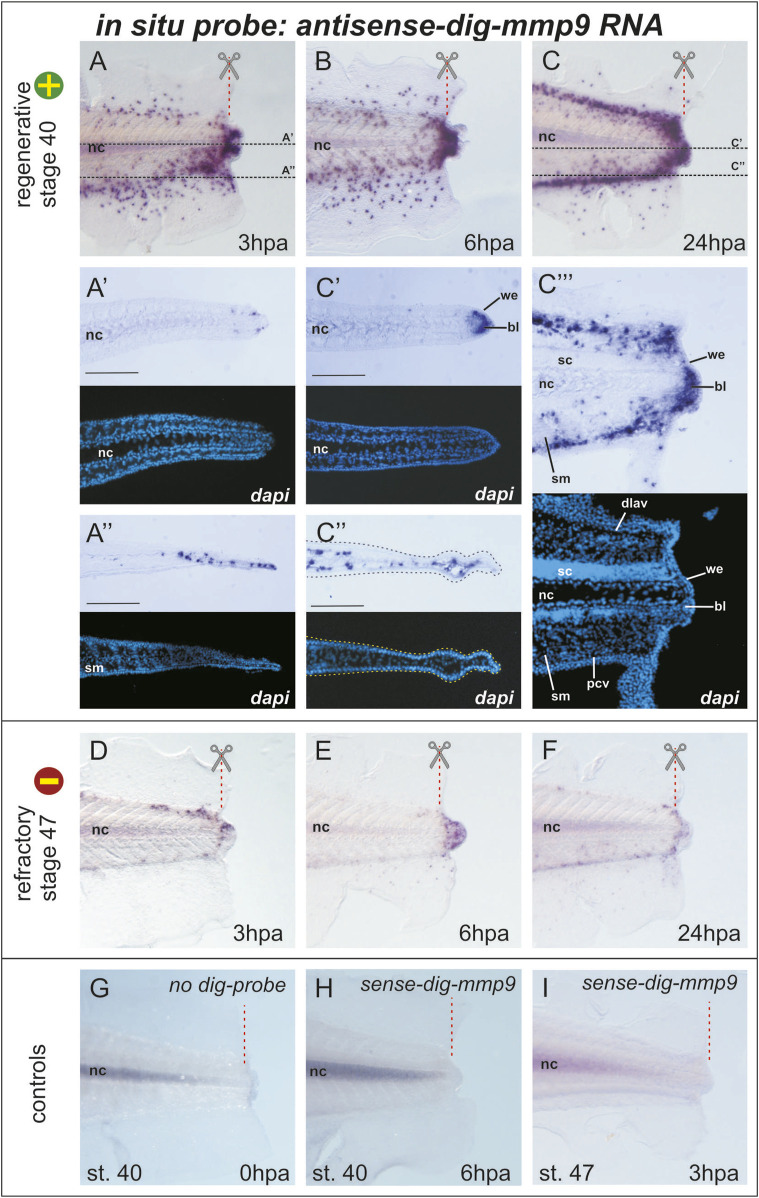
*Mmp9* expression pattern in the tail of *Xenopus laevis* tadpoles after amputation at stage 40 (regeneration competent) and stage 47 (regeneration incompetent) revealed by an optimized whole-mount *in situ* hybridization protocol followed by cryo-sectioning **(A–C)** Images of the distal part of the tail 3, 6, or 24 h post-amputation stained (blue-violet dots) for cells expressing *mmp9*. The red dashed line with scissors indicates the amputation line, and black dashed lines indicate the position of the frontal cryosections of **(A’, A”)** and **(C’, C”)**. Light microscopy images of sections are combined with DAPI fluorescence. Scale bar 200 µm **(C’’’)** The light microscopy image of the sagittal cryo-section of the tail at 24 hpa after *in situ* hybridization for *mmp9* expression with the corresponding DAPI fluorescence image. Bl–blastema, dlav–dorsal longitudinal anastomosing vessel, nc–notochord, pcv–posterior cardinal vein, sc–spinal cord, sm–somatic muscles, we–wound epithelium **(D–F)** *Mmp9* expression pattern in tadpole tails 3, 6, or 24 h after amputation during the refractory period (stage 47, regeneration incompetent) **(G–I)** Images of control *in situ* hybridization performed without dig-labeled RNA probe **(G)** or with sense-dig-labeled *mmp9* RNA probe show similar background staining of the notochord. The intensity of notochord staining was positively correlated with the duration of the chromogenic reaction. No positive signals were detected in other tissues of regenerating tail.

To clarify the data on the temporal-spatial distribution of cells with *mmp9* expression, we tested *mmp9* gene activity in tissues of the regenerating tail of *X. laevis* tadpoles using our new *in situ* hybridization protocol optimized for these samples. Using the fourth variant of treatments (see Figure 1), we performed *in situ* hybridization on tadpole tails amputated at stage 40 (regeneration-competent) or at refractory stages 46–47 (regeneration-incompetent) and fixed at 0, 3, 6, and 24 hpa (Figure 2). Samples for each time point (3, 6, 24 hpa, 12–15 tadpoles) were collected in three independent experiments. Images in Figure 2 show one tadpole out of ≥75% of tadpoles from each group at a given time point that have the same expression pattern of *mmp9* mRNA in cells of regenerating tails.

As shown in Figure 1, taken immediately after tail amputation, *mmp9*+ cells are predominantly scattered in the area of the dorsal and ventral tail fins. When comparing *mmp9* expression patterns in the regenerating tail at 0, 3, 6, and 24 hpa, a sharp increase in *mmp9*+ cells can be observed in the distal region of the caudal fins, as well as in the notochord and neural tube below the amputation line (Figures 1, 2A). The staining of the notochord in the proximal region of the tail is background, as it appears during hybridization both without RNA-probe and with the sense-RNA probe (compare Figures 2B, H). Considering that in early development, *mmp7*, *mmp9*, and *mmp18* are active in macrophages and are required for their migration (Tomlinson et al., 2008) new data on the *mmp9* expression pattern during regeneration are likely the result of active migration of myeloid cells towards the injury. At the same time, we cannot exclude the possibility that, in addition to the migration of *mmp9*+ cells, there is also an induction of *mmp9* expression in the cells of the tail stump. Furthermore, by 24 h post-amputation, the orderliness of migrating *mmp9*+ cells increases greatly, resulting in a concentration of these cells along the main vascular pathways. Notably, the temporal *mmp9* expression profile revealed here using WISH is consistent with the *mmp9* temporal profile obtained by other authors using bulk RNA-seq of the regenerating tail in the more rapidly developing species *Xenopus tropicalis*, in which the expression peak of this gene was observed as early as 15 hpa (Chang et al., 2017).

To determine in which tissues *mmp9*+ cells are localized, we made frontal and sagittal cryo-sections (20 µm thick) of tails stained by WISH. Figure 2 shows that at the level of the notochord, cells expressing *mmp9* appear near its distal tip, and their number increases significantly at 24 hpa, filling the space between the tip of the notochord and the wound epithelium. This area is usually defined as a blastema (Figures 2A’, C’). At first glance, it appears that the *mmp9*+ cells in the fin region lie in the outer epithelial layer. However, a frontal section at the level of the ventral caudal fin shows that *mmp9*+ cells are distributed underneath it in the stroma or vessels of the fin (Figures 2C’, C’’). Additionally, the sagittal section at 24 hpa demonstrates stained cells concentrated above and below the notochord and spinal cord. According to the literature, these cells correspond to the dorsolateral anastomosing vessel and posterior cardinal vein (Levine et al., 2003; Senevirathne et al., 2020). It is also clear that the closer the myeloid cells are to the distal end, the more actively they spread to the area of muscle tissue and the spinal cord (Figure 2C’’’).

*In vivo* tracing of labeled myeloid lineages, including granulocytes and macrophages, during wounding and infection in *Xenopus* tadpoles has shown that myeloid cells near the injury site start migrating as early as 20 min post-injury. The myeloid cells roll along the vascular endothelium or extravasate from vessels, migrating toward the wound area (Paredes et al., 2015). Mmp9 (gelatinase B) is a secreted matrix metalloproteinase that degrades proteins of the extracellular matrix, particularly collagen IV, VII, and X (Fu et al., 2009). The distribution of type IV collagen was detected at stage 56 in the basement membrane within the skin, between muscle cells, and around the spinal cord and notochord lamella (Nakajima and Yaoita, 2003). The detected pattern of the reparative myeloid marker *mmp9* during the first day after tail amputation can be explained by combining all these data. Myeloid cells, likely attracted by chemokines or other agents, move toward the damage (amputation line) along vessels and also between cells of tissues containing collagen IV, VII, or X, cutting a path through the ECM using mmp9 as a machete. Such active ECM disruption near the amputation line could induce the release of ECM-linked factors, alter the cellular connectome and signaling repertoire, stimulate cellular dedifferentiation for blastema formation, and provide spatial freedom for further proliferation.

The next question we addressed was whether and how the expression pattern of *mmp9* changes if the tail is amputated during stages when it is incompetent for regeneration, i.e., during the so-called refractory period (stages 45–47). Indeed, WISH staining of *mmp9* transcripts at 3, 6, and 24 hpa at refractory stages differed significantly from that at the regeneration-competent stage 40 (compare Figures 2A–F). As shown in Figures 2D–F, the number of *mmp9*+ cells in refractory tadpoles is catastrophically low beneath the amputation line as well as in the tail fin tissues. By 6 hpa, *mmp9*+ cells are concentrated in the tail tip, and by 24 hpa, the number of cells decreases. At 2 and 3 dpa, *mmp9* expression drops to zero levels, similar to 0 dpa (data not shown). These results suggest an interconnection between myeloid cell activity and regeneration progression.

Comparing the obtained results with scRNA-seq data, some inconsistencies in expression levels can be seen. The *mmp9* expression, as determined by the scRNA-seq transcriptomic atlas, is downregulated by 1 dpa (Aztekin et al., 2019), in contrast to the apparent upregulation detected using our optimized *in situ* hybridization protocol. The expression level of *mmp9* in the refractory period according to scRNA-seq data is higher than at stage 40, while we demonstrate the opposite results. Undoubtedly, the large volume of new omics data is indispensable and very useful for guiding research. However, validating the data using proven old-fashioned methods is still a necessary step. The obtained high-quality imaging data of regenerating tails stained for *mmp9*-expressing cells by the optimized WISH protocol made it possible to observe the life of these cells at the early stages of regeneration and to clarify and supplement the data obtained by high-throughput methods. It is likely that the proposed protocol will be suitable for addressing signal-to-noise ratio problems in similar samples, such as the regenerating fin of *Danio rerio*, the tail of *X. laevis* during normal development or the regenerating hindlimb, regenerating tail, or limb of *Ambystoma mexicanum*. In addition, we believe it is worth testing whether our additional sample treatments are applicable to variations of *in situ* hybridization, such as two-color WISH or fluorescent multichannel WISH (Long and Rebagliati, 2002; Vize et al., 2009).

## Data Availability

The original contributions presented in the study are included in the article, further inquiries can be directed to the corresponding author.
